# A RCT to explore the effectiveness of supporting adherence to nebuliser medication in adults with cystic fibrosis: fidelity assessment of study interventions

**DOI:** 10.1186/s12890-024-02923-z

**Published:** 2024-03-21

**Authors:** J. M. Bradley, M. Hutchings, M. A. Arden, A. O’Cathain, C. Maguire, M. J. Wildman, on behalf of the CFHealthHub Study Team

**Affiliations:** 1 Wellcome-Wolfson Institute for Experimental Medicine, School of Medicine, Dentistry and Biomedical Sciences, Queen’s University, Belfast, UK; 2 Sheffield Adult Cystic Fibrosis Centre, Sheffield Teaching Hospitals NHS Foundation Trust, Northern General Hospital, Herries Road, Sheffield, S5 7AU UK; 3 Centre for Behavioural Science and Applied Psychology, Sheffield Hallam University, Collegiate Crescent, Sheffield, S10 2BQ UK; 4 School of Health and Related Research, University of Sheffield, Regent Court, 30 Regent Street, Sheffield, S1 4DA UK; 5 Clinical Trials Research Unit, University of Sheffield, Regent Court, 30 Regent Street, Sheffield, S1 4DA UK

**Keywords:** Fidelity, Design, Training, Treatment fidelity, Receipt, Enactment

## Abstract

**Background:**

A multi-component self-management intervention ‘CFHealthHub’ was developed to reduce pulmonary exacerbations in adults with Cystic Fibrosis (CF) by supporting adherence to nebuliser medication. It was evaluated in a randomized controlled trial (RCT) involving 19 CF centres, with 32 interventionists, 305 participants in the intervention group, and 303 participants in the standard care arm. Ensuring treatment fidelity of intervention delivery was crucial to ensure that the intervention produced the expected outcomes.

**Methods:**

Fidelity of the CFHealthHub intervention and standard care was assessed using different methods for each of the five fidelity domains defined by the Borrelli framework: study design, training, treatment delivery, receipt, and enactment. Study design ensured that the groups received the intended intervention or standard care. Interventionists underwent training and competency assessments to be deemed certified to deliver the intervention. Audio-recorded intervention sessions were assessed for fidelity drift. Receipt was assessed by identifying whether participants set Action and Coping Plans, while enactment was assessed using click analytics on the CFHealthHub digital platform.

**Results:**

Design: There was reasonable agreement (74%, 226/305) between the expected versus actual intervention dose received by participants in the CFHealthHub intervention group. The standard care group did not include focused adherence support for most centres and participants. Training: All interventionists were trained. Treatment delivery: The trial demonstrated good fidelity (overall fidelity by centre ranged from 79 to 97%), with only one centre falling below the mean threshold (> 80%) on fidelity drift assessments. Receipt: Among participants who completed the 12-month intervention, 77% (205/265) completed at least one action plan, and 60% (160/265) completed at least one coping plan. Enactment: 88% (268/305) of participants used web/app click analytics outside the intervention sessions. The mean (SD) number of web/app click analytics per participant was 31.2 (58.9). Additionally, 64% (195/305) of participants agreed to receive notifications via the mobile application, with an average of 53.6 (14.9) notifications per participant.

**Conclusions:**

The study demonstrates high fidelity throughout the RCT, and the CFHealthHub intervention was delivered as intended. This provides confidence that the results of the RCT are a valid reflection of the effectiveness of the CFHealthHub intervention compared to standard care.

**Trial registration:**

ISRCTN registry: ISRCTN55504164 (date of registration: 12/10/2017).

**Supplementary Information:**

The online version contains supplementary material available at 10.1186/s12890-024-02923-z.

## Background

Treatment fidelity can be defined as “the extent to which an intervention is implemented as intended” [1]. Understanding treatment fidelity provides researchers with greater insight as to why an intervention has been successful. Understanding treatment fidelity also helps to confirm whether the intervention has accurately tested the hypothesis, that the results of the trial are attributable to the intervention, and that the intervention can be replicated. Failure to assess fidelity increases the risk of dismissing effective interventions that were unsuccessful due to poor implementation, or conversely, accepting ineffective interventions based on favourable results that were unrelated to the intervention [2–6]. This is particularly pertinent to complex interventions, which include several interacting components, where the mechanism of action is dependent on a number of different variables working in unison. Treatment fidelity is also relevant when trials are delivered across multiple sites by multiple providers, introducing potential variation in delivery and therefore effectiveness of the intervention both at the individual and site level [7]. Treatment fidelity ensures that the results of an intervention are directly attributable to the intervention and that no extra treatment factors have been included.

The CFHealthHub intervention was developed through a rigorous and systematic process detailed elsewhere [8]. It was evaluated in a 12-month RCT undertaken in 19 CF centres recruiting 608 participants randomised to the intervention (*n* = 305) and standard care (*n* = 303) [9]. The aim of the RCT was to investigate the effectiveness of a multi-component (complex) self-management intervention (CFHealthHub) compared with standard care in adults with Cystic Fibrosis (CF) using pulmonary exacerbation incidence rate as the primary outcome and adherence to prescribed nebuliser treatment as a key secondary outcome. The RCT found that pulmonary exacerbations did not show a statistically significant difference, but the CFHealthHub intervention achieved higher objectively measured adherence versus standard care [9].

The large number of centres (*n* = 19) and interventionists (*n* = 32) from a variety of disciplines were recruited to support the delivery of this study. We recognised that this could potentially increase the potential for variation in delivery between centres and impact on the clinical trial outcomes.

Briefly the intervention comprised a digital platform, accessed via the web or a smartphone application (app) which displays real time graphs and tables of objectively measured nebuliser adherence. This data formed the basis of conversations between the participant and a health professional employed to deliver the intervention within the RCT (interventionist). Modules of behaviour change techniques were built into CFHealthHub for either independent use by participants, or for use within an intervention session. These were designed to increase motivation for adherence, to address capability and opportunity barriers, and to build habits for treatment-taking.

Interventionists were supported with a comprehensive manual including procedures and worksheets to aid consistent intervention delivery. The content of the web platform and app was tailored to individual participants’ needs based on their nebuliser medication prescription, and their responses to a questionnaire which incorporated a modified version of the Beliefs about Medicines Questionnaire (BMQ-Specific) [10] to highlight beliefs about the perceived necessity and concern beliefs for nebuliser treatment, and to identify capability and opportunity barriers. Once the participant responses were added to CFHealthHub, specific modules of content were recommended for them, ensuring the selected information highlighted was specific to the individual. A range of techniques could be used to overcome individual motivation, capability and opportunity barriers, for example supporting people with CF to create habits for treatment, i.e. taking treatments in response to specific contextual cues can help to sustain adherence and to lower perceptions of treatment burden [11]. Therefore, key tools included Action Plans in which participants made during intervention sessions if–then plans [12] identifying a specific cue for treatment taking and linking this to a specific treatment dose, and Coping Plans or back-up plans [13] in which participants made plans to overcome specific barriers to their Action Plans. Interventionists were trained, and reminded via worksheet cues, for the plans to be patient led. Once plans had been created, and modules of CFHealthHub selected to meet the needs of the individual, the platform displayed this personalised information and plans in a 'Toolkit' area.

**Fig. 1:**
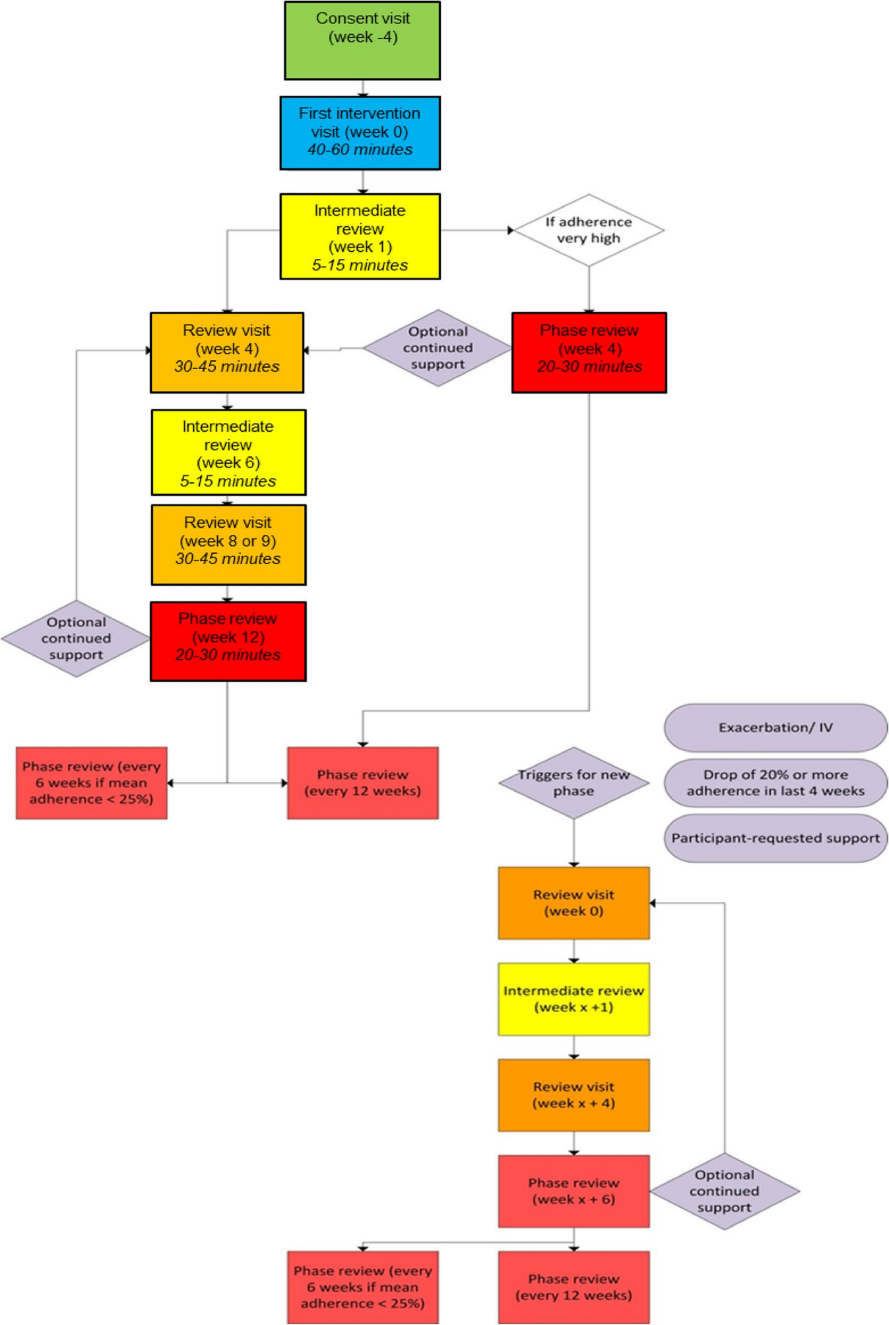
Schedule of intervention delivery. All participants received their first intervention visit at least 4 weeks following consent (so that the consultation is based on at least 4 weeks of adherence data). This visit was always done face-to-face including, hospital (in-patient), clinic, or home. All participants received an intermediate review phone call one week later. Subsequent visits depended on their adherence level. Participants with adherence of ≥ 80% followed the 'Very high adherence' pathway; those with adherence < 80% followed the normal pathway. Participants on the ‘Very high adherence’ pathway had intervention sessions over a 4-week period. In addition to the first intervention session (week 0) and an intermediate review (week 1) they received a phase review at week 4. They then received a phase review session every 12 weeks. Participants on the normal pathway had intervention sessions over a 12-week period. In addition to the first intervention session (week 0) and an intermediate review (week 1) they received a review session at week 4, an intermediate review at week 6, a second review session at weeks 8 or 9 and a phase review at week 12. This pattern of delivery constitutes a phase

Participants were supported to interact with the digital content and tools in sessions alongside the trained interventionists following a manualised delivery procedure, with a person-centred communication style. The intervention schedule is detailed in Fig. 1. All participants received a first intervention visit (face-to-face) and intermediate review phone call. Subsequent visits were determined based on the participant’s adherence level. Participants with an adherence level of ≥ 80% followed the 'Very high adherence' pathway while participants with adherence levels of < 80% followed the normal pathway. Participants could access the web/app-based platform outside formal intervention sessions and also had the option to receive regular notifications (e.g. informing them if they had achieved adherence goals or encouragement to increase adherence).

We recognised the importance of considering treatment fidelity in the design and delivery of the intervention delivered in the RCT. If delivered as intended, we hypothesised it had greatest likelihood of producing the outcomes. However, in practice, behavioural interventions are often delivered inconsistently resulting in variability in treatment outcomes. If fidelity to the interventions was found to be high, any significant effects found would be attributed to the intervention itself rather than the inclusion of unplanned components, thus avoiding the potential for an ineffective intervention being further researched or adopted in clinical practice. Conversely, if the intervention was found to be ineffective and fidelity was not attained this could result in a potentially effective intervention being disregarded.

We used the Borrelli Framework measurement tool of fidelity [7, 14] to assess, monitor, and enhance treatment fidelity across five domains (Supplementary Table 1).

### Aims

This paper aims to report on the assessment of fidelity under the five domains as set by the Borrelli Framework measurement tool of fidelity (Supplementary Table 1). In the context of fidelity, we assessed:

i. whether the participants received their assigned intervention as expected, i.e., CFHealthHub intervention or standard care intervention (Design domain)
ii. the interventionists knowledge, understanding and competency to deliver the CFHealthHub intervention (Training domain)
iii. the interventionists approach to the preparation of the CFHealthHub intervention; adherence to the content of the assigned interventions; the quality of the content for each type of intervention i.e., 1.^st^ intervention; review and phase review (Delivery domain)
iv. whether participants understood and performed treatment-related behavioural skills and cognitive strategies during treatment delivery (Receipt domain)
v. whether participants were able to enact these skills in real-life settings (Enactment domain)

## Methods

### Fidelity approach and understanding the intervention

The pilot study phase of the CFHealthHub RCT was used to refine the different methods of assessment for each fidelity domain [8, 15–17] (Supplementary Table 2). The different methods of assessment for each fidelity domain are described below.

### Design fidelity

This explored whether the groups received their assigned intervention as expected, i.e. CFHealthHub intervention or standard care intervention. For the CFHealthHub intervention group, there was complexity in the flow of intervention delivery where the next step in the intervention flow could change in response to events during the trial (Fig. 1). This complexity influenced the expected intervention dose. The minimum expected intervention dose was expressed using pre-determined cut-off criteria. The cut off criteria was set as 75% for ‘low’ and ‘high’ adherence and this included all patients who received the correct sessions/ in any ordering in the correct pathway (Fig. 1). Participants who did not follow a pathway and may never have had a phase review but may have had multiple review sessions were defined as receiving intervention sessions.

To explore if the patients assigned to the standard care group received standard care (which we defined for the purposes of the RCT as receiving no focused adherence support), we summarised the number of centres during the course of the RCT that used objective data such as medicines possession ratio (MPR) or iNeb adherence data to inform care. All centres were asked to complete a usual care survey at study start and at 12 months. This was an 11-item questionnaire which explored adherence support given at site to the standard care group during the study. Our rationale being that without having adherence data available they could not deliver focused adherence support. A brief description of the types of visits i.e. 1st intervention visit, review visit and phase review is provided (Supplementary Table 3). These descriptions are taken from the original report [18].

### Training fidelity

A programme of training was delivered to all interventionists that was supplemented throughout the duration of the trial. The compulsory training involved eight days of a combination of face-to-face and remote training, and it equipped the interventionists with the skills to deliver all three types of consultation (1st intervention visit, review visit and phase review visit).

Interventionists were also asked to complete evaluation forms at the end of training to rate their competence to deliver the intervention.

#### Competency assessment

Competency to deliver the CFHealthHub intervention as planned was assessed by the fidelity team in two ways:

1. Theoretical competency test: This competency test assessed knowledge of key elements of the intervention and was undertaken prior to practical training and practical competency tests. To be fully certified as competent to deliver the intervention, interventionists had to pass a theoretical competency test (≥ 80%). If a pass mark was not achieved, then feedback/additional training was provided and if required the part/whole of the test was repeated.
2. Practical competency tests: Certification to deliver the 1st intervention visit was conducted using a mock patient scenario (developed using real anonymised patient data from the CFHealthHub feasibility study [8]. Certification to deliver the review/ phase review visits was conducted as they occurred in the main RCT with a study participant. Where participants provided consent, intervention sessions were audio-recorded (Supplementary Appendix 2). We developed checklists to assess adherence to the content and quality of the intervention (Supplementary Tables 4–6) based on the completion of worksheets by the interventionist during delivery and the verbal interactions they had with participants (assessed during role play or audio-recordings of sessions). As recommended by Borrelli [7], interventionists had to achieve ≥ 90% in the practical competency assessment to be deemed certified to deliver the intervention. Borrelli [7] recommended this high score as deterioration in skills post training is common. Those achieving < 90% were given individual feedback and tutoring as well as direction to specific learning materials and mock role-play. Interventionists were given three opportunities for certification. If they did not meet the standards for certification after this point, they were mentored one-to-one during the course of the trial until they could demonstrate independent competence at delivering the intervention.

### Treatment delivery fidelity

#### Treatment delivery of intervention group

All fidelity assessments were conducted by two fidelity assessors independently. Agreement between fidelity assessors scoring was assessed. After independent assessment, the two fidelity assessors discussed scoring and reached consensus on final scores.

The process for recording and uploading visit data by interventionists for fidelity assessment is outlined in Supplementary Appendix 2. We had clear criteria to inform choice of audio-recorded intervention sessions for the assessment of treatment delivery over the course of the trial (fidelity drift). To facilitate the assessment of fidelity drift, a standard report was generated regularly (every 2 weeks) from study data held centrally in a variety of sources. Using criteria for targeted and random assessment data as described in Table 1, the fidelity assessment team selected and assessed intervention sessions for fidelity and aimed to complete fidelity assessments within 2 weeks. Although some drift in quality is unavoidable across the lifespan of the study, a significant level of quality needed to be obtained to ensure the CFHealthHub intervention was delivered as planned. Therefore, the pass mark for the practical competency assessment of fidelity drift was lower but still set at a high level. Interventionists were required to achieve ≥ 80% in assessments for fidelity drift; if the interventionist achieved < 80%, they were offered additional individual feedback and tutoring. Results of these assessments were discussed by the fidelity assessment team, and any common areas for improvement were identified and informed weekly teleconferences with interventionists. Booster training sessions were provided if the fidelity drift assessments identified further training was needed.

**Table 1:** Detail of targeted and random assessment of fidelity drift

We pragmatically aimed to sample at least 20% of interventions for drift. Drift was assessed by sampling a total 213 of different types of visits (1st intervention visit, review visit or phase review visit). We had set criteria to inform the sampling for assessment of drift:	
Targeted assessment	• All interventionists who failed any certification assessment (ie 1st intervention, 1st Review, 1st Phase review visit), • Interventionists with high withdrawal rates (more than two participants withdrawn from interventionist contact) • Interventionists with in-sufficient number of audio recordings (less than 80% audio recorded out of those who provided consent) • Interventionists where CFHealthHub data indicated a lower than expected number of visits completed, and/or action and coping plans created. It is noted that as action/coping plans are central to the delivery of this behaviour the inclusion of these was reviewed regularly and targeted retraining delivered even before drift was assessed formally
Random assessment	• If the targeted assessment was less than 10% of all interventionist visits, random assessment of interventionists / sites took place in order to bring the total sample of assessments up to 10% of all interventionist visits

#### Treatment delivery of standard care group

The standard care arm used eTrack data-logging Controllers for adherence data collection, but participants and care teams had no access to the adherence data. Contamination was also minimised since there was no access to the CFHealthHub intervention, behavioural change tools and content.

### Receipt fidelity

To assess fidelity receipt, we focused on aspects of the CFHealthHub intervention for which we had accurate records of actions taken by participants during intervention sessions. We therefore operationalised receipt by assessing whether participants had set action and coping plans to increase their treatment adherence at the 1st intervention visit and at review visits. These plans were recorded by the participant within the CFHealthHub digital platform.

### Enactment fidelity

To assess fidelity enactment, we focused on aspects of the CFHealthHub intervention interaction by participants independently i.e. occurred outside of intervention delivery sessions. This was determined by ascertaining the proportion of participants that interacted with some component of CFHealthHub either via the web or the app and the average number of times the interaction happened. We also explored the number of participants who enabled push notifications or reminders, for example, push notification to indicate when they had achieved an adherence goal or encouragement to increase adherence.

### Analysis

We specified our analyses in the Statistical Analysis Plan (SAP) [19].

#### Design

We used descriptive statistics to assess the proportion of participants who received the expected intervention dose of the CFHealthHub intervention. For the usual care survey, medians and IQRs were used to summarise questionnaire items and change scores were calculated to estimate changes over the time period of the study.

#### Training

We used descriptive statistics to assess the proportion of participants who passed the competency assessments.

#### Treatment delivery

We used descriptive statistics to assess the proportion of participants who passed the fidelity drift assessments. As interventionists were rated independently by two assessors, fidelity scores were compared between assessors using Bland Altman plots [20] and an intraclass correlation coefficient was calculated.

Overall fidelity was calculated at centre and study level using the following methodology which took into account that fidelity assessment occurred at multiple times through the trial, and that these differed by interventionist. A score remained valid until a subsequent fidelity assessment occurred.

Weighted score for interventionist = Σ score x weight

$$\mathrm{Weight}\left(\text{w}\right)=\frac{\mathit n\mathit u\mathit m\mathit b\mathit e\mathit r\mathit\;\mathit o\mathit f\mathit\;\mathit d\mathit a\mathit y\mathit s\mathit\;\mathit s\mathit c\mathit o\mathit r\mathit e\mathit\;\mathit i\mathit s\mathit\;\mathit v\mathit a\mathit l\mathit i\mathit d}{\mathit t\mathit o\mathit t\mathit a\mathit l\mathit\;\mathit d\mathit a\mathit y\mathit s}$$

$$\mathrm{Centre}\;\mathrm{mean}=\frac{\sum Weighted\;score}n$$

To summarise the fidelity assessment process, a line graph showing overall fidelity scores by interventionists over time was plotted. The timing of assessments and training was indicated with symbols and threshold scores (90% at certification and 80% during the study).

#### Receipt

We used descriptive statistics to assess the proportion of participants who used action and coping plans.

#### Enactment

We used descriptive statistics to assess the proportion of participants who used click analytics and the mean number of click analytics used over the duration of the study.

## Results

### Sample description

The number of interventionists at each site (alongside their professional background) delivering the intervention are summarised in Supplementary Table 7. Primary outcome data were available for all participants; adherence data were missing for only 3% (19/608) of participants.

### Design fidelity

#### Intervention group

Table 2 details the number of participants who completed the CFHealthHub intervention as expected, calculated using a range of cut-offs to define expected intervention dose. In general, there was reasonable agreement (74%) between expected intervention dose versus actual intervention in the treatment group.

**Table 2:** Participants who completed expected sessions

	**Participation**	
	**(n)**	**(%)**
Minimum intervention dose:	226/305	74.10
Identifies the number of participants by site and overall who received the correct sessions/minimum, in any ordering, in the correct pathway with 75% as the cut off between ‘low’ and high’ adhererence		
75–80% per protocol:	172/305	56.40
Identifies the number of participants by site and overall who received the correct sessions, in the correct order, in the correct pathway. This calculation recognised that participants in the range of 75–80% could choose to go in either low or high adherence pathways based on their perceived need for support		
1st and phase review:	237/305	77.70
Identifies the number of participants by site and overall who received a minimum of a 1st and phase review session. This essentially identifies anyone who received the minimum sessions in either the low or high adherence pathway		
2 sessions:	276/305	90.50
Identifies the number of participants by site and overall who received a minimum of 2 sessions which includes any of 1st, review, phase review		
3 sessions:	259/305	84.90
Identifies the number of participants by site and overall who received a minimum of a 3 sessions which includes any of 1st, review, phase review		

#### Standard care

Participants in the standard care arm had not seen their adherence data or other parts of the intervention, indicating contamination was low. The usual care survey confirmed that there were no changes in adherence consultations during the study period (Supplementary Table 8). Adherence conversations and collection and utilisation of adherence data varied in frequency and formality; many sites reported using objective measures on an ad hoc or relatively infrequent basis. No other type of adherence consultations were used during the study period (< 10% used objective data such as medicines possession ratio (MPR) or iNeb adherence data to inform care) and no other objective graphs were used within centres to chart adherence.

### Training fidelity

In total, there were there were 32 interventionists across 19 sites recruited. However, not all interventionists completed all components of the training due to the timing of their recruitment onto the study team. 30 interventionists completed the theoretical competency test. This was not completed for 2 interventionists that joined later. 5 interventionists did not complete the practical competency related to the first intervention visit as they joined the intervention team after all first intervention visits were completed. 2 interventionists did not complete practical competency assessments for review and phase review intervention visits.

#### Theoretical competency test

Thirty interventionists completed the theoretical competency test. 27/30 interventionists achieved the pass mark (≥ 90%) on first attempt. Three interventionists did not pass the theoretical competency test (scores 80, 72, and 73) due to a lack of depth and breadth to their answers. They were given additional mentoring to ensure full understanding and subsequently passed on the second attempt.

#### Practical competency test

27/32 interventionists had first intervention visits assessed and were certified. 27/32 interventionists completed the mock 1st intervention competency assessment prior to conducting any actual interventions in the RCT (agreed pass score ≥ 90%). The mean (SD) final score was 96 (4) %, range, 88 to 100%. At the first intervention visit, 26/27 interventionists were assessed once before certification and oral feedback was given to interventionists on any areas that lost marks. 1/27 interventionist failed for reasons including use of language not in line with the spirit of the intervention, missing content, approach to action and coping planning. Following one-to-one mentoring and re-education, this interventionist passed this assessment on the second attempt.

For review and phase review visits, 30 interventionists were assessed and certified. At the review visit, 9/30 interventionists failed the first assessment. 8/9 passed following a second session and 1/9 passed following a third session. For review assessments, the mean (SD) final score was 96.2 (3.7), range, 90.7 to 100%. For the phase review visit, 6/30 interventionists failed the first assessment and 5/6 passed following a second session and 1/6 passed following a third session. For the review assessments and phase review was 96 (3.2), range, 91.7 to 100%. Interventionists who failed were given individualised retraining before reassessment and were subsequently certified. Fidelity scores are summarised for each type of assessment in Table 3.

**Table 3:** Intervention fidelity delivery score summaries by session type for certification and fidelity drift

**Session type**	**Assessment**^**a**^		**N**	**Mean (SD)**	**Median (IQR)**
First intervention visit	**Certification**	1st assessment	27	96.0 (3.8)	97.2 (92.3, 100.0)
		Re-assessment	1	98.6 (-)	98.6 (98.6, 98.6)
	**Fidelity Drift**	Assessment	29	94.1 (8.1)	95.8 (93.1, 97.2)
Review Visit	**Certification**	1st assessment	30	89.8 (12.3)	92.6 (87.0, 98.1)
		Re-assessment	9	94.6 (4.0)	96.3 (94.4, 96.3)
	**Fidelity Drift**	Assessment	47	91.5 (8.7)	92.6 (90.2, 96.3)
Phase Review Visit	**Certification**	1st assessment	30	92.7 (9.1)	94.4 (91.7, 97.2)
		Re-assessment	6	93.2 (10.3)	97.2 (93.1, 99.3)
	**Fidelity Drift**	Assessment	34	92.7 (7.9)	94.4 (91.7, 97.2)

^a^Reasons for assessment, with multiple reasons possible: certification (97), reassessment after failed certification (36), high withdrawal rate (18), insufficient audio-recorded sessions (37), fewer than expected intervention visits or action/coping plans created (82), random to ensure total assessment sample ≥ 20% of all interventionist visits (9)

Evaluation forms also demonstrated that interventionists rated their competence to deliver the CFHealthHub intervention at the end of the training as high (mean: 7.6 on a 10-point Likert scale). There were 5 different waves of training during the trial and there was no difference in how interventionists rated competence between each of the waves of training (Wave 1, 7.1; Wave 2, 8.3; Wave 3, 6.8; Wave 4, 8.1).

### Treatment delivery fidelity

#### Treatment delivery of intervention group

Two assessors independently assessed each intervention session and agreement between assessors was high. Intra-class correlation coefficients were as follows: Assessors 1 & 2, 0.93 (0.87, 0.96); Assessors 1 & 3 0.84 (0.76, 0.89); Assessors 2 & 3, 0.90 (0.85, 0.94). Of all paired assessments during the trial (213 in total) there was 97.2% agreement (207 of 213 assessments in agreement) when comparing pass/fail decisions at the 80% threshold.

**Fig. 2:**
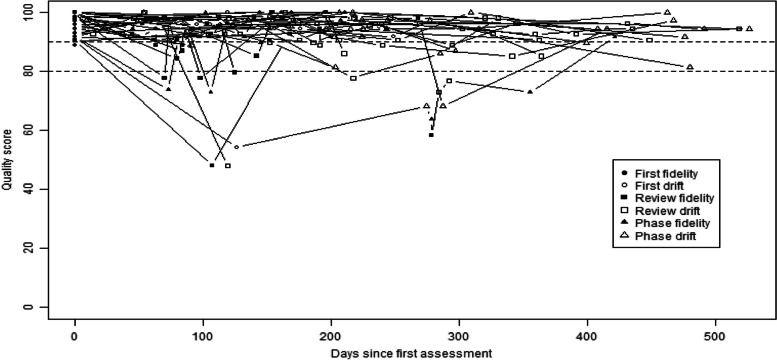
Interventionist quality scores over the course of the assessment period

**Fig. 3:**
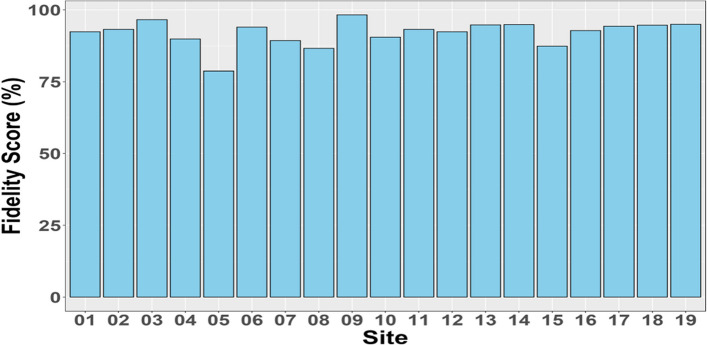
Overall fidelity scores by site

Individual interventionists’ fidelity scores over the course of the RCT are shown in Fig. 2. Each assessment score was weighted by the time for which it was valid and means calculated by interventionist, then aggregated by site. Overall fidelity quality scores by site are provided in Fig. 3. The trial had good fidelity (overall fidelity by site range 79–97%) with only one site not achieving the mean threshold (> 80%) on fidelity drift assessments.

For the fidelity drift phase, the maximum number of reassessments per stage was two. All reassessed interventionists received a ‘booster’ training session. One interventionist did not achieve the pass mark threshold of 80% in drift assessment. Scores for fidelity drift were lower than for certification showing small levels of fidelity drift which indicated adequate quality of intervention delivery.

#### Treatment fidelity for standard care

The usual care arm, and interventionists did not have access to CFHealthHub and there was no activity to suggest otherwise from the click analytics data.

### Receipt fidelity

Of the participants that completed the 12-month CFHealthHub trial, 205/265 (77%) completed at least one action plan and 160/265 (60%) completed at least one coping plan.

### Enactment fidelity

One hundred ninety-five participants agreed to receive notifications via the mobile app. The mean (SD) number of notifications per participant was 53.6 (14.9).

268/305 (88%) participants used web/app click analytics outside the intervention sessions. The mean (SD) number of click analytics per participant was 31.2 (58.9). Additionally, qualitative data reported elsewhere provided evidence that participants were able to enact skills in real life settings [21].

## Discussion

This paper demonstrates the implementation of the processes in applying fidelity framework to a complex behavioural change intervention delivered in a multicentre RCT. In the CFHealthHub intervention and standard care intervention, we were able to successfully measure fidelity across the domains of design, training, delivery, and receipt and demonstrated high fidelity across these domains. It was harder to measure enactment but there was evidence that some enactment occurred. There is discussion in the literature regarding the value of fidelity assessment alongside the main outcome evaluation [6, 22]. Assessment and reporting of fidelity domains across broad behavioural interventions has been shown to be variable and whilst generally high in terms of design (80%), it has been shown to be relatively low across the other domains of training (22%), delivery (35%), receipt (49%) and enactment (57%) [7]. We have demonstrated how use of frameworks such as Borrelli [7], can improve fidelity assessments within trials.

In trials, especially when complex interventions are delivered to some patients whilst other patients receive standard care at the same site, the opportunity for contamination may be significant. Recognising the potential for contamination and being able to demonstrate that no contamination occurred provides confidence that the observed outcomes are related to proposed treatment mechanisms rather than the presence or absence of unmeasured “active” ingredients in the standard care intervention.

Many trials, even those considered straight forward now require more than generic skills from researchers and often involve more complex behaviour, for example, capturing data, dose regulation based on previous results and using more complex technologies and equipment, therefore bespoke training and delivery is becoming more relevant to a broader array of studies. Despite this, few trials formally assess the competence of the interventionists to deliver the intervention or maintain competence during the trial. Future trials with complex interventions, delivered across multiple sites or different treatment pathways should consider a process or framework such as the Borrelli framework [7] to ensure that staff are formally trained and certified and that competency is maintained during the trial and with staff turnover.

Our focus in this trial was ensuring high fidelity to the assigned interventions and ensuring that this was maintained throughout the trial. To that end we monitored and then intervened if we identified concerns with regards to fidelity. More broadly, there is a balance and choice to be considered in fidelity assessment: to assess ‘naturally occurring’ fidelity at the end of a trial or to monitor and intervene to ensure high fidelity throughout the trial.

### Strengths and limitations

We successfully implemented a fidelity assessment into a large complex trial and were able to analyse fidelity results in real time in order to optimise fidelity across sites and throughout the timeline of the study and consequently can be confident that the results of this trials can be attributed to the intervention. The methodology to facilitate fidelity assessment was rigorous, all regulatory approvals were in place at the beginning of the trial and “fidelity staff” were available for competency assessment and retraining throughout the trial.

Borrelli proposed that up to 50% of interventionist should be assessed [7]. However, there are no clear guidelines to inform the optimum criteria (proportion of drift in the literature that should be assessed or indeed the methodology around choice of intervention for drift) on which to base the sampling frame for assessment of drift. We pragmatically aimed to sample at least 20% of interventions for drift. In the majority of cases more than one month had lapsed between the interventionists’ certification and their delivery of the 1st intervention to a participant, so all interventionists were required to refresh themselves about the intervention to ensure there was no deterioration in skills.

While some aspects of fidelity are easier to measure, measuring receipt and enactment was more challenging. We defined receipt in terms of how many participants completed action/coping plans and enactment in terms of how many participants used web/app click analytics outside of the intervention. However, these measurements did not include all aspects of receipt and all aspects of enactment and are therefore only indicators of these types of fidelity. We recommend future trials clearly consider the measures and methods that they will use to assess fidelity and pilot these early on in the process.

When funding streams are limited, fidelity assessment is often seen as less of a priority. We recommend that fidelity assessments should be considered as essential for complex trials and adequate funding and resources allocated for this work stream; if fidelity is not assessed and maintained, it is unclear if the results are directly attributable to the intervention.

Whilst treatment fidelity monitors and enhances the validity and reliability of interventions, it does not directly assess the acceptability of the intervention to the participants or to the interventionists/ healthcare providers. Acceptability was measured in this study and was reported elsewhere [18]. Future trials should consider relevance of frameworks such as The Theoretical Framework of Acceptability [23] to reflect the extent to which people delivering or receiving a healthcare intervention consider it to be appropriate.

## Conclusions

This study demonstrates the practical application of Borrelli’s fidelity framework [7] and measurement tools to assess fidelity of the CFHealthHub intervention within an RCT. This study demonstrates that fidelity was high throughout the RCT and therefore provides confidence that the results of the RCT are a valid reflection of the effectiveness of the CFHealthHub intervention compared to standard care.

### Supplementary Information

**Supplementary Material 1. **

## Data Availability

The datasets used and/or analysed during the current study are available from the corresponding author on reasonable request.

## Abbreviations

CF: Cystic Fibrosis
RCT: Randomised Controlled Trial
BMQ-Specific: Beliefs about Medicines Questionnaire
MPR: Medicines possession ratio
SAP: Statistical analysis plan
