# Supplementary material for: A RCT to explore the effectiveness of supporting adherence to nebuliser medication in adults with cystic fibrosis: fidelity assessment of study interventions
Source: BMC Pulm Med. 2024 Mar 21;24:148. doi: 10.1186/s12890-024-02923-z (PMC10956306; doi:10.1186/s12890-024-02923-z)
Supplement: Supplementary file 1 — Supplementary Material 1. [file 12890_2024_2923_MOESM1_ESM.docx]

**Intervention training and treatment fidelity**

J.M. Bradley (PhD)^1^, M. Hutchings (BSc)^2,^ M.A. Arden (PhD)^3^, A. O’Cathain (PhD)^4^, C. Maguire (MPH)^5^, M.J. Wildman (PhD)^2,4^ on behalf of the CFHealthHub Study Team

Supplemental Material

**Supplementary Appendix 1** Members of the CFHealthHub Study Team

**Supplementary Appendix 2** Supplementary Methods

**Supplementary Table 1** NIH BCC Consortium Treatment Fidelity Checklist with the methodologies used in CFHealthHub trial

**Supplementary Table 2** Refinement of fidelity assessment procedures during the feasibility study and implications for the main RCT

**Supplementary Table 3** Descriptions of study visits

**Supplementary Table 4** CFHealthHub Fidelity Assessment First Intervention – For Certification

**Supplementary Table 5** CFHealthHub Fidelity Assessment Review - For Certification

**Supplementary Table 6** CFHealthHub Fidelity Assessment Phase Review- For Certification

**Supplementary Table 7** Interventionists at each site and their professional background

**Supplementary Table 8** Sites responses to usual care survey at study start and at 12 months (excluding free text questions).

**Supplementary Appendix 1** Members of the CFHealthHub (CFHH) Study Team

Martin J Wildman^2,4^, Alicia O’Cathain^4^, Daniel Hind^5^, Chin Maguire^5^, Madelynne A Arden^3^, Marlene Hutchings^2^, Judy Bradley^1^, Stephen J Walters^4^, Pauline Whelan^6^, John Ainsworth^6^, Paul Tappenden^4^, Iain Buchan^6,7^, Rachel Elliot^8^, Jon Nicholl^4^, Stuart Elborn^1^, Susan Michie^9^, Laura Mandefield^4^, Laura Sutton^4^, Zhe Hui Hoo^2,4^, Sarah J Drabble^4^, Elizabeth Lumley^4^, Daniel Beever^3^, Aline Navega Biz^4^, Anne Scott^5^, Simon Waterhouse^5^, Louisa Robinson^5^, Mónica Hernández Alava^4^, Alessandro Sasso^4^

**Supplementary Appendix 2** Supplementary Methods

Process for recording and uploading visit data for fidelity assessment

The interventionist was instructed to audiotape the intervention and check that the audio file was encrypted. This encrypted file was then uploaded to a site-specific folder on google drive and then downloaded by the Clinical Trials Research Unit (CTRU). The encrypted audio file was deleted from google drive as soon as it was downloaded and checked. CTRU stored the files on the secure University server and these were available to the fidelity assessment team. The interventionists completed all session report forms on paper case report file (CRF) and emailed/ posted copies to CTRU. The interventionists also completed an intervention session log on Prospect (the electronic data management system). These audiotapes and accompanying intervention assessment worksheets were assessed independently by two assessors against the fidelity checklist. These checklists have been specifically developed for certification of interventionists in the CFHH intervention trial. The assessors discussed their individual scoring and agreed a fidelity score by teleconference. Both individual assessor scores and the final agreed score was logged on a local, shared spreadsheet. Additionally, a final agreed fidelity assessment sheet was sent to CTRU to enter onto Prospect.

**Supplementary Table 1** NIH BCC Consortium Treatment Fidelity Checklist with the methodologies used in CFHealthHub trial

| Treatment fidelity strategy | CFHealthHub intervention |
| --- | --- |
| **Treatment design**  1. Treatment dose in the intervention condition(s)  Length of contact session(s) | One ‘first intervention’ visit, 40–60 minutes; two ‘intermediate’ reviews, 5–15 minutes each; two ‘main’ reviews, 30–45 minutes each; one ‘phase’ review, |
| Number of contacts | Dependent on levels of adherence as outlined in Figure 1. |
| Content of treatment | Multi-component self-management intervention including a web platform and app with real time adherence data and includes modules of behaviour change techniques and education tools designed to increase motivation for adherence. |
| Duration of contact over time | As outlined in Figure 1, participants on the normal pathway (adherence <80%) had intervention sessions over a 12-week period. In addition to the first intervention session (week 0) and an intermediate review (week 1) they received a review session at week 4, an intermediate review at week 6, a second review session at weeks 8 or 9 and a phase review at week 12. This pattern of delivery constitutes a phase. They then receive a phase review session every 12 weeks, or 6 weeks if their adherence is <25%.  Participants on the ‘very high adherence’ pathway (adherence ≥80%) had intervention sessions over a 4-week period. In addition to the first intervention session (week 0) and an intermediate review (week 1) they received a phase review at week 4. They then received a phase review session every 12 weeks. |
| 2. Treatment dose in the control or comparison condition  Length of contact session | N/A |
| Number of contacts | N/A |
| Content of treatment | Standard care (no focused adherence support) |
| Duration of contact over time | N/A |
| 3. Provider credentials | Health care professionals/ interventionists |
| 4. Theoretical model or clinical guidelines | Theoretical Domains Framework |
| 5. Potential confounders that limit the ability to make conclusions | Fidelity drift of intervention during the trial |
| 6. Plans to address possible setbacks in implementation | Training, certification and fidelity drift assessment and re-training and booster training during the trial |
| 7. If more than one intervention, describe all equally well  **Training providers**  1. How providers were trained | N/A  Eight days of training to deliver all three types of consultation (1^st^ intervention visit, review visit and phase review visit). |
| 2. Standardization of provider training | Standardized training sessions and training materials delivered to all interventionists. |
| 3. Measurement of provider skill acquisition post training (pre-field implementation) | Interventionists were required to complete (a) theoretical competency assessment as part of training and (b) practical competency assessments for 1^st^ intervention visit using a mock patient scenario and for review/ phase review visits using real patients as part of the main RCT. |
| 4. How provider skills were maintained | Fidelity drift assessments, re-training and booster training during the trial. |
| 5. Characteristics being sought in a treatment provider | Health care professionals |
| 6. Fit between the provider and the intervention at the hiring stage | Physiotherapists, nurses, psychologists, pharmacists, dietitian |
| **Delivery of treatment**  1. Method to ensure that the content of the intervention was being delivered as specified | Review of audio recordings using checklists |
| 2. Method to ensure that the dose of the intervention was being delivered as specified | Analysis of expected vs actual dose |
| 3. Mechanism to assess if the provider actually adhered to the intervention plan | Review of audio recordings using checklists |
| 4. Evaluation of non-specific treatment effects (eg perceived warmth) | Review of audio recordings using checklists |
| 5. Treatment manual | Intervention manual available |
| 6. Plan for assessment of whether active ingredients were delivered | Review of audio recording using checklists |
| 7. Plan for assessment of whether proscribed components were excluded | Review of audio recording using checklists |
| 8. Plan for how will contamination between conditions be prevented | To explore if the control group received standard care, the number of centres during the course of the trial that used objective data such as medicines possession ratio (MPR) or iNeb adherence data to inform care were summarised. |
| 9. A priori specification of treatment fidelity | To be certified, interventionists must achieve ≥90%  For fidelity drift, interventionists must achieve ≥80% |
| 10. Post-activation adaptations or modifications in treatment delivery | None |
| **Receipt of treatment**  1. Assessing subject comprehension of the intervention during the intervention period | Use of action/coping plans |
| 2. Strategy to improve subject comprehension of the intervention | Additional support dependent on adherence levels |
| 3. Assessing subject ability to perform the intervention skills during the intervention period | Additional support dependent on adherence levels |
| 4. Strategy to improve subject performance of intervention skills during the intervention period | Additional visits dependent on adherence levels |
| 5. Multi-cultural factors considered in the development and delivery of the intervention | Some sites had different cultural alignment with the intervention |
| **Enactment of treatment skills**  1. Subject performance of the intervention skills assessed | Use of web/app click analytics and qualitative interviews. |
| 2. Strategy to improve subject performance of the intervention | Additional support dependent on adherence levels |

Table adapted from Salloum et al. Reporting Treatment Fidelity in Behavioral Tobacco Treatment Clinical Trials: Scoping Review and Measurement Recommendations. *Nicotine Tob Res* 2022; 24: 150–159.

**Supplementary Table 2** Refinement of fidelity assessment procedures during the feasibility study and implications for the main RCT

| **Methods** | **Key findings** | **Implications for main RCT** |
| --- | --- | --- |
| Face to face training was delivered to these three interventionists in these sites over 1.5 days with further training delivered online. | “Communication with patient and optimising patient participation” was highlighted as a specific area for improvement in interventionists’ skills.  Assessment of fidelity of intervention delivery indicated that not all of the active ingredients of the intervention were being adequately delivered and there was feedback that the online training was difficult to complete during recruitment. The worksheets were also quite difficult to follow. | An additional module was created to cover this in training for the main RCT.  The manual was revised, training and worksheets to focus more on the fidelity of the intervention and the quality of the delivery. |
| Fidelity checklists were developed by reviewing the session report forms, to explore what the interventionists were meant to do during the visit. Criteria relating to the items in the preparation of visits and delivery stages of visits were extracted and formulated into a Fidelity Checklist. The team agreed on the criteria for the quality of how these items should be delivered. |  | In relation to the checklist, additional items were added to preparation content and the quality content section to ensure a comprehensive review of all aspects of fidelity. |
| 3 members of the fidelity team independently assessed each consultation. | There was good consensus among the 3 assessors. | 2 rather than 3 assessors of fidelity scored the interventions independently and then agreed a consensus score for fidelity. |
| Each interventionist delivered the intervention to 16 patients. Interventionists were asked to audiotape and choose one of the first five Visit 1 interventions to submit for assessment by the fidelity assessment team. These consultations were planned to be used to pilot the fidelity checklists for skill acquisition (certification) and also skill maintenance (drift). | There were key issues with the timing of submission of the consultations, audio tapes, and completed worksheets by the interventionists that did not support the timely assessment of fidelity and this impacted on our ability to conduct any drift assessment within the feasibility study. | Provisions were made within the protocol; for example, interventionists must submit the audiotape and completed worksheets for all interventions to the CRTU in real time. |
| Three interventionists were trained to deliver the intervention in six face-to-face meetings over 5 months to 32 participants who had access to the CFHH website throughout | Less intervention sessions had been delivered than intended | We therefore devised a clear intervention pathway that indicated the frequency, interval and pattern of intervention sessions for each participant in the main RCT |
| 32 participants had access to the CFHH website throughout | Participants showed only very limited engagement with the CFHH web platform in between intervention sessions. | The CFHH mobile app development was prioritised for the main RCT. Tools were added to encourage engagement i.e. push notifications sent from the app each Monday congratulating participants on meeting their target or encouraging them to engage if they had not accessed the platform for 2 weeks. |

**Supplementary Table 3. Descriptions of study visits**

| **Type of consultation** | **Definition** |
| --- | --- |
| **1^st^ intervention visit** | This session always happens face-to-face, in a hospital/clinic setting or at home.  It is the first time that the participant accesses the CFHealthHub platform and sees their data. The session is individualised to the participant but in general covers modules linked to treatment, self-monitoring, confidence building and goal setting, treatment plan and problem-solving. |
| **Review visit** | This was usually delivered face-to-face or by telephone. The session focuses on the data and adherence since the last visit.  The precise focus varied depending on the individual participant.  The session covered the following modules linked to treatment, self-monitoring, confidence building and goal setting, treatment plan and problem-solving. |
| **Phase review visit** | The focus of this appointment was to reflect on and to consider whether continued support is required or whether the participant wished to manage their adherence independently. Ideally this should be delivered face-to-face but can be delivered by telephone.  The Phase Review visit covered the following modules:   - My treatment - Self-monitoring - Confidence building - Problem-solving |

Supplementary Table 4

| **Summary of Scores (visit 1)** | |
| --- | --- |
| Intervention – Adherence to Content | __ % |
| Preparation – Adherence to Content | __% |
| Intervention –  Quality of Content | __% |
| Average Scores > 90% - meets criteria for certification  Average Scores < 90% - areas of improvement required | |

| **First Intervention – Preparation – Adherence to Checklist** |  |
| --- | --- |
| **Y = 1 N = 0 (please circle)** |  |
| Review com-BBQ responses (problems / barriers) | **0 / 1** |
| Have up to 3 modules of My Treatment been included in My Toolkit | **0 / 1** |
| Addition of videos only for treatments prescribed | **0 / 1** |
| Review Motivation/Confidence | **0 / 1** |
| Review Adherence chart tables | **0 / 1** |
| Review weekly adherence for last 4 weeks | **0 / 1** |
| **Total** | **__ / 6**  **(__%)** |

| **First Intervention**  **additional modules identified, if relevant.ection X)one view; traffic light; target line; hover over times; date slider)Intervention - Adherence to Content** |  |
| --- | --- |
| **Y = 1 N = 0 (please circle)** |  |
| CF HealthHub Introduction | **0 / 1** |
| Review of My Toolkit | **0 / 1** |
| Review of motivation rating | **0 / 1** |
| Review adherence graphs/charts/orientation (key views=one view; traffic light; target line; hover over times; date slider) | **0 / 1** |
| Review adherence and factors affecting adherence success | **0 / 1** |
| Review patterns of treatment | **0/1** |
| Review factors affecting non-adherence | **0 / 1** |
| Create action plan | **0 / 1** |
| Create target goal | **0 / 1** |
| Discussion on problems / solutions (solutions= coping plan; party planner; problem solving) | **0 / 1** |
| Discussion around confidence | **0 / 1** |
| Review of session Scheduled next appointment (date/time) | **0 / 1** |
| **TOTAL** | **_ /12**  **(__%)** |

**CFHealthHub Fidelity Assessment First Intervention – For Certification**

| **First Intervention – Quality of Content** | | |
| --- | --- | --- |
| Quality delivery CFHealthHub introduction | 0  1  2 | 0 = Poor-missing  1 = Overly long does not follow script / Does not encourage participant to navigate  2 = Good- brief orientation follows script and encourages patient to navigate |
| Quality explanation around My Toolkit | 0  1  2 | 0 = Poor- missing  1 = Navigates key areas but not made clear that content is personalised, or some other components missing  2 = Good- navigates key areas emphasises personalised content, describes modules from my treatment and locates/opens one module |
| Quality of discussion on motivation rating | 0  1  2 | 0 = Poor- no discussion / judgemental discussion  1 = Discussion but limited listening/reflection  2 = Good-listening and reflection evident |
| Quality of review of adherence graphs / charts / orientation (key views=one view; traffic light; target line; hover over times; date slider) | 0  1  2 | 0 =Poor -did not show any key views/ functions  1=Some key views/functions missed, or overly long time spent describing  2=Good- all key views/functions covered |
| Quality of discussion factors, affecting  adherence successes. | 0  1  2 | 0=Poor missing/lack of focus on success.  1=Limited discussion on factors that affect success, not participant-led.  2=Good-Discussion on factors affecting success, led by participant and reflected back by interventionist. |
| Quality of discussion on patterns of treatment | 0  1  2 | 0 = Poor. missing  1 = Discussion but limited listening/reflection  2 = Good - listening and reflection evident |
| Quality of discussion on factors affecting non-adherence. | 0  1  2 | 0= Poor missing/lack of focus on factors affecting adherence.  1=Limited discussion on factors that affect adherence, not participant-led.  2=Good-Discussion on factors affecting adherence factors led by participant and reflected back by interventionist. |
| Quality of discussion on action planning | 0  1  2 | 0=Poor-missing key instructions and no identification of if/when cues.  1=Instructions present and if/when cues identified, but not participant-led.  2=Good- clear instructions and appropriate if/ when cues identified, participant-led. |
| Quality of discussion on target goal | 0  1  2 | 0= Poor-did not discuss/gave target goal with no participant participation.  1= Some discussion with participant but goal not participant led  2= Gooddiscussion and participant-led goal agreed |
| Quality of discussion on problems / solutions (solutions= coping plan; party planner; problem solving) | 0  1  2 | 0 = Poor- no solutions to key problems/barriers or instruction on what to do  1 = Some solutions/strategies missed to address key problems and/or lack of negotiation.  2 = Good- participant led approach to finding solutions for key problems. |
| Quality of discussion around confidence | 0  1  2 | 0 = Poor- no discussion/ judgemental/ closed questions.  1 = Discussion but limited listening/ reflection.  2 = Good- listening/ open questions/ reflection evident and reminder of help/ support/ additional modules identified, if relevant. |
| Quality of review of session | 0  1  2 | 0= Poor-missing/not done.  1= Some key components missed.  2= Good- all key components reviewed and message about learning included |
| TOTAL | __ /24 | **­­__%** |

**CFHealthClub Fidelity Assessment First Intervention – Version 1 12.02.2018**

| **Site …………………………………………**  **Interventionist ……………………………** |
| --- |
| **Assessor 1 ………………………………..**  **Average Quality Score = ……..%**  **Assessor 2 ………………………………..**  **Average Quality Score = …… %** |
| **CONSENSUS QUALITY SCORE ……%** |

**CFHealthHub Fidelity Assessment Review - For Certification**

Supplementary Table 5

| **Pre- Review – Preparation – Adherence of Checklist** |  |
| --- | --- |
| **Y = 1 N = 0 (please circle)** |  |
| Review adherences chart tables | **0 / 1** |
| Review of goal whether goal met or not met | **0 / 1** |
| **Total** | **__ / 2**  **(­­__%)** |

| **Summary of Scores (Review visit)** | |
| --- | --- |
| Preparation – Adherence to Checklist | __ % |
| Intervention – Adherence to Content | __% |
| Intervention –  Quality of Content | __% |
| Scores > 90% - meets criteria for certification  Scores < 90% - areas of improvement required | |

| **Visit Review – Intervention Adherence to Content** |  |
| --- | --- |
| **Y = 1 N = 0 (please circle)** |  |
| Review of additional browsable areas on CF healthhub | **0 / 1** |
| Review adherence charts/tables | **0 / 1** |
| Review of factors affecting success | **0 / 1** |
| Review of factors affecting nonadherence | **0 / 1** |
| Review of motivation rating | **0 / 1** |
| Review action plan | **0 / 1** |
| Review target goal | **0 / 1** |
| Discussion on problems /barriers/ solutions (solutions= coping plan; party planner; problem solving) | **0 / 1** |
| Discussion around confidence | **0 / 1** |
| Review of modules in modules included in My Toolkit (maximum of 3) | **0 / 1** |
| Review of session | **0 / 1** |
| Scheduled next appointment (except last appointment) | **0 / 1** |
| **TOTAL** | **__ / 12**  **(__%)** |

| **Visit Review – Quality of Content** | | |
| --- | --- | --- |
| Quality of review of additional browsable areas on CF healthhub  (Section X) | 0  1  2 | 0= Poor- missing  1= Limited discussion but not interactive  2= Good-interactive discussion of current content and potential additional useful content |
| Quality of discussion on factors affecting success  (Section X) | 0  1  2 | 0=Poor missing/lack of focus on success  1=Limited discussion on factors that affect success, not participant led  2=Good-Discussion on factors affecting success, led by participant reflected back by interventionist |
| Quality of discussion on factors, affecting nonadherence  (Section X) | 0  1  2 | 0= Poor missing/lack of focus on factors affecting adherence  1=Limited discussion on factors that affect adherence, not participant led  2=Good-Discussion on factors affecting adherence factors led by participant reflected back by interventionist |
| Quality of discussion on motivation rating  (Section X) | 0  1  2 | 0 = Poor- no discussion / judgemental discussion  1 = Discussion but limited listening/reflection  2 = Good-listening and reflection evident |
| Quality of discussion on action planning  (Section X) | 0  1  2 | 0=Poor-missing key instructions and no if/when cues  1=Instructions present and if/when cues, but not participant led  2=Good- clear instructions and appropriate if/when cues, participant led. |
| Quality of discussion on target goal  (Section X) | 0  1  2 | 0= Poor-did not discuss/gave target goal with no participant participation  1= Some discussion with participant but goal not participant-led  2= Good-and participant led goal agreed |
| Quality of discussion on problems/barriers / solutions (solutions= coping plan; party planner; problem solving)  (Section X) | 0  1  2 | 0 = Poor- no solutions to key problems/barriers or instruction on what to do  1 = Some solutions/strategies missed to address key problems and/or lack of negotiation  2 = Good- participant led approach to finding solutions for key problems. |
| Quality of discussion around confidence  (Section X) | 0  1  2 | 0 = Poor- no discussion /judgemental/closed questions  1 = Discussion but limited listening/reflection  2 = Good- listening/reflection evident and reminder of help/support/additional modules identified if relevant |
| Quality of review of session  (Section X) | 0  1  2 | 0= Poor-missing/not done  1= Some key components missed  2= Good- all key components reviewed and message about learning included |
| TOTAL | __ / 18 | __% |

**ACtiF Fidelity Assessment Visit ReviewCerification– Version 1. 20/06/2016**

**Assessor 1 ……………………………………**

Average quality score =……… %

**Assessor 2 …………………………………...**

Average quality score =………. %

**CONSENSUS QUALITY SCORE = %**

**Site ……………………….**

**Interventionist ……………………………..**

**CFHealthHub Fidelity Assessment - Phase Review For Certification**

Supplementary Table 6

| **Phase Review – Intervention Adherence to Content** |  |
| --- | --- |
| **Y = 1 N = 0 (please circle)** |  |
| Review Goal (when goal met, adherence high effort) | **1**  **0** |
| Review adherence (success / factors affecting adherence success) | **1**  **0** |
| Review perceived benefits of adherence | **1**  **0** |
| Review motivation and confidence | **1**  **0** |
| Confirm target goal | **1**  **0** |
| Review independent use of CFHH | **1**  **0** |
| Review triggers for future contact. | **1**  **0** |
| **TOTAL** | **__ / 7 (__%)** |

| **Phase Review – Preparation – Adherence of Checklist** |  |
| --- | --- |
| **Y = 1 N = 0 (please circle)** |  |
| Review Recent Adherence/ data in charts/ tables | **1**  **0** |
| Review weekly adherence figures | **1**  **0** |
| Review goal lowest/ highest adherence in a week | **1**  **0** |
| **Total** | **__ / 3 (__%)** |

| **Summary of Scores** | |
| --- | --- |
| Preparation – Adherence to Checklist | __ % |
| Intervention – Adherence to Content | __% |
| Intervention –  Quality of Content | __% |
| **Scores > 90% - meets criteria for certification**  **Scores < 90% - areas of improvement required** | |

CONSENSUS QUALITY SCORE=…………%

Assessor 1……………………………………..

Average Quality Score=…………%

Assessor 2…………………………………….

Average Quality Score……………………

**Site ……………………….**

**Name ……………………………..**

| **Phase Review – Quality of Content** | | |
| --- | --- | --- |
| Quality of discussion on achieving goals since last visit. | **0**  **1**  **2** | **0 = Poor (missing discussion when goal met.**  **1 = Limited discussion on positives eg when goal met, adherence high, good effort.**  **2 = Good – discussion focused on positives with clear identification when goal met, adherence high, good effort.** |
| Quality of discussion on adherence (success,  factors, affecting adherence) | **0**  **1**  **2** | **0 = Poor missing/lack of focus on success.**  **1 = Limited discussion on factors that affect success, not participant-led.**  **2 = Good-Discussion on factors affecting success, led by participant and reflected back by interventionist.** |
| Quality of discussion on perceived benefit of adherence. | **0**  **1**  **2** | **0 = Poor. No discussion/ judgemental discussion.**  **1 = Discussion but limited listening/ reflection.**  **2 = Good listening/ reflection on benefits.** |
| Quality of discussion on target goal | **0**  **1**  **2** | **0 = Poor. Missing or target goal set without discussion**  **1 = Some discussion but goal not participant led**  **2. = Good. Discussion and goal participant led** |
| Quality of discussion on independent use of CFHealthHub | **0**  **1**  **2** | **0 = Poor – missing.**  **1 = Navigates key areas but some components missing.**  **2 = Good. Navigates key areas. Emphasises how to use independence and make changes.** |
| Quality of discussions on triggers for future contact | **0**  **1**  **2** | **0 = Poor – missing.**  **1 = Limited/ does not check understanding/ satisfaction.**  **2 = Good/ clear explanation and understanding/ satisfaction checked.** |
| **TOTAL** | **__ / 12**  **(__ %)** |  |

**CFHealthHub Fidelity Assessment Phase Review – Version 1. 12.02.2018**

**Supplementary Table 7** Interventionists at each site and their professional background

| **Site** | **Profession** | | | | | |
| --- | --- | --- | --- | --- | --- | --- |
|  | **CF Physiotherapist*** | **CF**  **Nurse** | **CF Pharmacy** | **CF Dietitian** | **Non-CF**  **Psychologist** | **Total at site** |
| **1** | 0 | 0 | 1 | 0 | 0 | 1 |
| **2** | 0 | 3 | 0 | 1 | 0 | 4 |
| **3** | 1 | 0 | 0 | 0 | 0 | 1 |
| **4** | 0 | 1 | 0 | 0 | 0 | 1 |
| **5** | 2 | 0 | 0 | 0 | 0 | 2 |
| **6** | 1 | 0 | 0 | 0 | 1 | 2 |
| **7** | 1 | 0 | 0 | 0 | 0 | 1 |
| **8** | 1 | 0 | 0 | 0 | 0 | 1 |
| **9** | 1 | 0 | 0 | 0 | 0 | 1 |
| **10** | 2 | 1 | 0 | 1 | 0 | 4 |
| **11** | 0 | 2 | 0 | 0 | 0 | 2 |
| **12** | 1 | 0 | 0 | 0 | 0 | 1 |
| **13** | 1 | 0 | 0 | 0 | 0 | 1 |
| **14** | 1 | 1 | 0 | 0 | 0 | 2 |
| **15** | 2 | 0 | 0 | 0 | 0 | 2 |
| **16** | 0 | 1 | 0 | 0 | 0 | 1 |
| **17** | 0 | 0 | 0 | 0 | 1 | 1 |
| **18** | 1 | 0 | 0 | 0 | 0 | 1 |
| **19** | 1 | 0 | 0 | 0 | 2 | 3 |
| **Total** | **16** | **9** | **1** | **2** | **4** | **32** |

*1 non-CF Physiotherapist

**Supplementary Table 8** Sites responses to usual care survey at study start and at 12 months (excluding free text questions).

| **Question** | **Study Start** | **12 months** | **Change** | **Interpretation** |
| --- | --- | --- | --- | --- |
| 1. Do you use MPR^a^ to understand a patient’s adherence during consultations? | - n = 19 - Median 5 (IQR 4–5) - 58% = never - 21% = rarely - 21% = sometimes | - n = 20 - Median 3.5 - (IQR 3.0–4.0) - 20% = never - 30% = rarely - 35% = sometimes - 15% = very often | - n = 19 - Median change in score 1 (IQR 0–2) - 21% = less frequent - 21% = no change - 58% = more frequent | On average, sites never used MPR during consultations at baseline. Use of MPR was more frequent for 58% of sites at follow-up |
| 2. Do you reduce the target prescription^b^ to promote adherence? | - n = 19 - Median 3 (IQR 3–4) - 5% = always - 16% = very often - 53% = sometimes - 21% = rarely - 5% = never | - n = 20 - Median 3 (IQR 2–4) - 40% = very often - 30% = sometimes - 30% = rarely | - n = 19 - Median change in score 0 (IQR 0–1) - 16% = less frequent - 47% = no change - 37% = more frequent | On average, sites would sometimes reduce a target prescription to promote adherence at baseline. This was largely consistent with the approach used at follow-up, although 37% of sites reported using this technique more frequently |
| 4. How often do you ask about/discuss adherence with your patients? | - n = 19 - Median 1 (IQR 1–2) - 63% = always - 32% = very often - 5% = sometimes | - n = 20 - Median 1.5 - (IQR 1.0–2.0) - 50% = always - 40% = very often - 10% = sometimes | - n = 19 - Median change score 0 (IQR –1 to 0) - 32% = less frequent - 53% = no change - 16% = more frequent | On average, sites always discussed adherence with their patients at baseline. There was a trend to discuss adherence less frequently in some sites at follow-up |
| 5. How important would you consider adherence support in your centre? | - n = 19 - Median 1 (IQR 1–2) - 63% = very important - 26% = important - 5% = moderately - 5% = slightly important | - n = 20 - Median 1 (IQR 1–2) - 65% = very important - 20% = important - 10% = moderately - 5% = slightly important | - n = 19 - Median change score 0 (IQR 0–0) - 16% = less important - 68% = no change - 16% = more important | On average, adherence support was considered to be very important at both baseline and follow-up |
| 6. In your centre which are priorities about improving CF? Please number 1–7, with 1 being the most important:  6(1) encouraging airway clearance  6(2) early treatment  of exacerbations with IVAB  6(3) encouraging exercise  6(4) early detection and treatment of diabetes  6(5) adherence to inhaled therapies (nebulised antibiotics)  6(6) adherence to inhaled therapies (mucolytics)  6(7) nutritional support to maintain BMI at target | - n= 13^c^ - Median by items 1–7: - question 6(1) – 2 (IQR 1–4) - question 6(2) – 4 (IQR 1–6) - question 6(3) – 6 (IQR 4.5–6.0) - question 6(4) – 3 (IQR 2–4) - question 6(5) – 3 (IQR 2–4) - question 6(6) – 4 (IQR 3–5) - question 6(7) –5 (IQR 3.5–6.0) - Of the sites that responded to this question, two annotated to say that all are actually priorities and one reported that this varied by patient and their individual clinical need | - n=17^d^ - Median by items 2, 3, 4 and 7: - question 6(2) – 4 (IQR 1–4) - question 6(3) – 7 (IQR 5–7) - question 6(4) – 5 (IQR 3–6) - question 6(7) – 4 (IQR 2–6) - n = 16* - Median by items 1, 5 and 6: - question 6(1) –2.5 (IQR 1–4.5) - question 6(5) – 3 (IQR 2.00–4.75) question 6(6) –3.5 (IQR 2.25–5.75) - One site responded with multiple items rated as ‘2’, indicating equal importance (these scores were not in the analysis) | - Not applicable | On average, encouraging airway clearance was considered the most important; encouraging exercise was considered the least important. There was substantial  variability  between sites in responses to this question and many felt that they could not answer because they felt that these practices were of equal importance and their priority varied by patient |
| 8. Do you use the  I-neb data to provide objectively recorded recent adherence data to inform your consultations (e.g. the percentages calculated per week or per month from the I-neb download)? | - n = 14 - Median 3 (IQR 2.75–5.00) - 7% = always - 14% = very often - 36% = sometimes - 14% = rarely - 29% = never | - n = 17 - Median 3 (IQR 2–5) - 12% = always - 18% = very often - 29% = sometimes - 12% = rarely - 29% = never | - n = 14 - Median change score 0 (IQR –0.25 to 1) - 21% = less frequent - 50% = no change - 29% = more frequent | An additional three sites acquired the use of I-nebs in usual care at follow-up. On average, sites sometimes used objective data downloaded from the I-neb to inform their consultations and this was largely consistent with practice at follow-up |
| 9. Do you use bespoke graphs plotted from the I-neb device that you have developed at your centre? | - n = 14 - Median 5 (IQR 5–5) - 93% = never - 7% = sometimes | - n = 17 - Median 5 (IQR 5–5) - 82% = never - 6% = rarely - 6% = sometimes - 6% = always | - n = 14 - Median 0 (IQR 0–0) - 7% = less frequent - 79% = no change - 14% = more frequent | The majority of sites at baseline (93%) or follow up (82%) never used bespoke graphs plotting I-neb data |
| 10. Do you sit with the patient and use Insight Online (the Philips graphical plotter) [Koninklijke Philips N.V.]? | - n = 14 - Median 5 (IQR 3–5) - 64% = never - 21% = sometimes - 7% = very often - 7% = always | - n = 17 - Median 5 (IQR 3–5) - 53% = never - 12% = rarely - 24% = sometimes - 12% = always | - n = 14 - Median 0 (IQR –1.0 to 0.5) - 29% = less frequent - 50% = no change - 21% = more frequent | On average, sites never used Insight Online with their patients at baseline or follow-up continued |
| 11. Do you have confidence that the percentage adherence from Insight Online is derived from the correct prescription (i.e. that it is based on the correct denominator)? | - n = 11 - Median 3 (IQR 2–5) - 9% = always - 36% = very often - 18% = sometimes - 36% = never | - n = 12 - Median 3 (IQR 2–4) - 33% = very often - 33% = sometimes - 17% = rarely - 17% = never | - n = 9 - Median 0 (IQR –1 to 2) - 33% = less frequent - 33% = no change - 33% = more frequent | Some sites felt this question was not applicable to them if they did not use I-neb data. Sites indicated that they sometimes had confidence in the adherence calculation derived from the prescription input. However, this varied between sites and between baseline and follow-up |

^a^ The number of prescriptions issued compared with the number cashed.

^b^ For example, agree that a patient with chronic Pseudomonas will aim to achieve 1 × DNase and 1 × tobramycin per day rather than 2 × tobramycin per day.

^c^ Sites that did not respond stated that they could not rank these items because they are all considered to be important.

^d^ Sites that did not respond stated that they could not rank these items as different patients have different priorities.

Coding questions 1, 2, 4 and 8–11: 1 = always; 2 = very often; 3 = sometimes; 4 = rarely; 5 = never.

Coding question 5: 1 = very important; 2 = important; 3 = moderately important; 4 = slightly important; 5 = not important.

Question 6: answers indicate order of priority [e.g. a question 6(1) score of three means that encouraging airway clearance was scored as third most important].
